# The impact of caries status on supragingival plaque and salivary microbiome in children with mixed dentition: a cross-sectional survey

**DOI:** 10.1186/s12903-021-01683-0

**Published:** 2021-06-25

**Authors:** Xiaoxia Yang, Lidan He, Siqi Yan, Xinyi Chen, Guoying Que

**Affiliations:** Stomatological Hospital, Southern Medical University, Guangzhou, 510280 China

**Keywords:** PacBio sequencing, Microbial community, Caries, Microhabitat, First permanent molar, Saliva, Deciduous tooth

## Abstract

**Background:**

Supragingival plaque and saliva are commonly used for microbiome analysis. Many epidemiological studies have identified deciduous teeth caries as a risk factor for caries development in first permanent molar (FPM); nevertheless, to the best of our knowledge, there are no reports on the effects of deciduous teeth caries on the microbiome of healthy FPM. Additionally, it remains unclear whether saliva can be used instead of supragingival plaque for caries microbial studies. Therefore, we aimed to elucidate this issue, and to characterize and compare the oral microbiome of healthy FPMs in children with different caries statuses and that from children with and without caries in a similar microhabitat, by PacBio sequencing. Currently, few studies have investigated the oral microbiome of children using this technique.

**Methods:**

Thirty children (aged 7–9 years) with mixed dentition were enrolled; 15 had dental caries, and 15 did not. Supragingival plaques of deciduous molars and maxillary FPMs, and non-stimulating saliva samples were collected. DNA was extracted and the v1–v9 regions of 16S rRNA were amplified. Subsequently, PacBio sequencing and bioinformatic analyses were performed for microbiome identification.

**Results:**

The microbial alpha diversity of the saliva samples was lower than that of the supragingival plaque (*p* < 0.05); however, no differences were detected between deciduous teeth and FPMs (*p* > 0.05). In addition, the alpha and beta diversity of children with and without caries was also similar (*p* > 0.05). Nonmetric multidimensional scaling and Adonis analyses indicated that the microbial structure of salivary and supragingival plaque samples differ (*p* < 0.05). Further analysis of deciduous teeth plaque showed that *Streptococcus mutans*, *Propionibacterium acidifaciens*, and *Veillonella dispar* were more abundant in children with caries than in those without (*p* < 0.05); while in FPMs plaque, *Selenomonas noxia* was more abundant in healthy children (*p* < 0.05). No differences in microorganisms abundance were found in the saliva subgroups (*p* > 0.05).

**Conclusion:**

We have determined that supragingival plaque was the best candidate for studying carious microbiome. Furthermore, *S. mutans*, *V. dispar*, and *P. acidifaciens* were highly associated with deciduous teeth caries. *S. noxia* may be associated with the abiding health of FPM; however, this requires additional studies.

**Supplementary Information:**

The online version contains supplementary material available at 10.1186/s12903-021-01683-0.

## Background

Caries, defined as the localized damage to the hard tooth tissue caused by acidic byproducts of bacterial fermentation of free sugars, is one of the most common, nonetheless preventable diseases in children [1–3]. It does not only affect the masticatory function, aesthetics, and school performance of children, but also reduces their quality of life [4]. Moreover, caries progression can cause pain [5] and potentially life-threatening complications, such as odontogenic brain abscesses [6, 7] and deep neck-space infections [8]. Once cavitated caries occurs, dental destruction progresses and becomes irreversible. In this sense, the American Associations for Dental Research has reported that untreated caries in deciduous teeth is the 10th-most prevalent condition, affecting 621 million children worldwide [9].

The biological basis of caries is the alteration of the microbial community, i.e., imbalance of the oral microbiome [10, 11]. Acidogenic and aciduric bacteria become abundant in a low-pH environments [12], and these mixed-species communities can cause caries via acid production, firm biofilm formation, and demineralizing of the tooth enamel. Several studies based on the 16S rRNA gene sequencing have demonstrated that children with caries have a different oral microbiome than healthy children [13–16]. However, while these results were obtained using next-generation sequencing (NGS) technology, which is a high-throughput and low-cost approach, it yields relatively short reads [17]. Therefore, it is insufficient for sequencing the complete 16S rRNA gene (total length: ~ 1500 bp), and can only sequence the integrant V region of the 16S rRNA gene. Accordingly, Teng et al. found that the selection of the specific V region affected the results of oral microbial diversity profiling [18].

Third-generation sequencing (TGS) platforms, such as sequencers of Pacific Biosciences (PacBio), have greatly improved these limitations. Although, the PacBio RS sequencer, released in 2011, had an average error rate of 13% [17, 19], higher than that of the NGS platforms, the subsequent development of the PacBio RSII and PacBio Sequel sequencers greatly improved the accuracy of these type of platforms [20]. The accuracy was enhanced through circular consensus sequencing (CCS) to 99.9999%, being now similar or higher than that of NGS. In addition, Myer et al. found that the TGS platform (PacBio RSII) achieved a better phylogenetic resolution than the NGS platform (MiSeq) [21]. However, only a few studies have analyzed the oral microbiome using PacBio sequencing, i.e., Wang et al. used the PacBio RSII sequencer to assess salivary microbial communities in children [22] and Ihara et al. studied the microbial communities of dental plaques from young adults with the PacBio Sequel sequencer [23]. To date, no study has investigated the microbiome from multiple regions of the oral cavity of children using PacBio sequencing.

Saliva and supragingival plaque on tooth surface are the most used samples to study caries. Nevertheless, whether saliva can be used instead of supragingival plaque to study caries remains controversial. Additionally, children with mixed dentition present primary and permanent teeth, and both cross-sectional and longitudinal studies have shown that caries of deciduous teeth is a risk factor for the caries development in first permanent molar (FPM) [24–26]. Nonetheless, to the best of our knowledge, there are no studies assessing the characteristics of the microbial community of healthy FPMs in children with and without caries in their deciduous teeth.

This study therefore set out to profile the microbiome present in supragingival plaque and saliva of children with different caries status, by PacBio sequencing. The specific aims of this research were, firstly, to evaluate potential optimal samples for caries analysis, and compare the microbiome of children with and without caries. Secondly, to determine whether the microbial community of healthy FPMs varies depending on the caries status of the deciduous teeth; and, finally, to compare the microbiome of the three types of oral samples collected (supragingival plaque of deciduous molars and maxillary FPMs, and non-stimulating saliva).

## Methods

### Subjects

For this study, 30 children (19 male and 11 female), 7–9 years of age, with mixed dentition were enrolled. All subjects were recruited from the same primary school in Guangzhou, China, to reduce the impact of environmental factors, such as fluoride concentration in drinking water, on the microbiome. The FPMs of all children had fully erupted without decay cavities, and deep pits and fissures were observed. The exclusion criteria in this study included: presence of a different oral disease, active bacterial or viral infections in other parts of the body, having received antibiotic treatment in the past 3 months, and usage of a removable or fixed orthodontic appliance.

The diagnostic criteria for caries were based on the fifth edition of the Oral Health Surveys: Basic Methods published by World Health Organization [27]. According to the results of oral examination, 15 children without caries were enrolled in the healthy group (H group), an 15 children with caries were enrolled in the caries group (C group). Additionally, the decayed, missing, and filled tooth (dmft) index and the decayed, missing, and filled tooth surface (dmfs) index were used to record caries status in all children. The dmft score of the H group was 0 (dmft = 0), indicating that they did not have caries; whereas that of the C group was ≥ 3, because this individuals presented caries in three or more deciduous teeth (dmft ≥ 3). The examination and sampling were performed by Xiaoxia Yang, who had previously been trained in the diagnosis of caries and appropriate sampling procedures.

### Sample collection

The children were not allowed to brush their teeth the night before nor in the morning of the sampling day, and water and food were withheld prior to sampling. Supragingival plaque was scraped with a sterile metal excavator and placed in a sterile Eppendorf tube containing 0.5 mL Tris–EDTA buffer. Dental plaque from the buccal and lingual surfaces and the caries lesions in deciduous molars was collected from individuals with caries (CD subgroup), and dental plaque from the buccal, lingual, and occlusal surfaces of deciduous molars was collected from healthy individuals (HD subgroup). The dental plaque collected from the buccal, lingual, and occlusal surfaces of FPMs of children from the C and H groups was labelled CP and HP subgroup, respectively. Two milliliters of non-stimulating saliva was collected from the bottom of the oral cavity of each child with a disposable sterile pipette and then placed in a sterile centrifuge tube. The saliva samples from the C and H groups were classified as CS and HS subgroups respectively). Every subgroup contained 15 samples; thus, a total of 90 samples were included in the study. Following collection, samples were placed in a foam incubator with dry ice, immediately transported to the laboratory, and stored at − 80 °C. The FPMs of all subjects were treated with pit and fissure sealant after sampling.

### DNA isolation and amplification

The total bacterial DNA was extracted using E.Z.N.A. Bacterial DNA Kit (Omega, USA) according to the manufacturer's protocol. DNA quality was evaluated via agarose gel electrophoresis and DNA concentration was assessed at 260 using a Nanodrop spectrophotometer (Thermo Scientific, USA), the absorbance ratios A260/A280 and A260/A230 were also determined to evaluate purity. Total DNA was stored at − 80 °C until use.

The barcode sequence was inserted into the forward primer (5′-AGAGTTTGATCCTGGCTCAG-3′) to distinguish each sample; additional details about the barcode sequences are shown in Additional file 1: Table S1. The v1–v9 regions of the 16S rRNA gene were amplified in a 25-μL amplification system containing 5 μL 5 × reaction buffer, 5 μL 5 × GC buffer, 2 μL dNTPs, 1 μL forward primer, 1 μL reverse primer (5′-GGTTACCTTGTTACGACTT-3′), 2 μL DNA template, 8.75 μL ddH_2_O, and 0.25 μL Q5 high-fidelity DNA Polymerase (NEB, USA). The PCR conditions were as follows: initial denaturation at 98 °C for 2 min, 25–30 cycles of denaturation at 98 °C for 15 s, annealing at 55 °C for 30 s, extension at 72 °C for 30 s, and a final extension at 72 °C for 5 min. The PCR products were examined using 2% agarose gel electrophoresis and extracted with an AxyPrep DNA Gel Extraction kit (Axygen, USA). The Quant-iT PicoGreen dsDNA Assay Kit (Thermo Fisher, USA) was used to quantify the obtained PCR products on a microplate reader (BioTek, USA). Based on the fluorescence quantitative results, the products from each sample were adjusted to equal concentrations and then mixed.

### DNA library construction and sequencing

DNA library was constructed using the SMRTbell Express Template Prep Kit 2.0 (Pacific Biosciences, USA), and each constructed library was combined with a primer and DNA polymerase to form a primer/template/polymerase, using the DNA Polymerase Binding Kit P4 (Pacific Biosciences). All complexes were loaded onto the SMRT Cell using the MagBead kit (Pacific Biosciences), and the MagBead was combined with the complex to travel over the zero-mode waveguide (ZMW). Sequencing was performed using the DNA Sequencing Kit 2.0 (Pacific Biosciences) on a PacBio Sequel Sequencer (Pacific Biosciences). The biotin-modified DNA polymerase bound to streptavidin at the bottom of the ZMW, anchoring the complex. The DNA polymerase read the single-stranded circular DNA template several times to produce polymerase sequences.

### Quality filtration of sequences

Several subreads were obtained by removing sequencing adapters from one polymerase sequence. The CCS approach was used to read subreads for at least three fully passes, thus producing CCS sequences with predicted accuracy of 99% and allocating the CCS sequences into the corresponding samples according to the barcode sequence. The QIIME (v1.8.0) software [28] was used to filter low-quality sequences that met the following conditions: sequence length below 500 bp; presence of one or more fuzzy base N; primer of 5′ terminal of sequence with more than 5 mismatched bases; and presence of more than 8 of the same continuous bases. USEARCH (v5.2.236) [29] via the QIIME (v1.8.0) software was used to check and eliminate chimeric sequences. Finally, high-quality sequences for each sample were obtained and used in subsequent analysis.

### The 16S rRNA sequence analysis

The sequence alignment tool UCLUST [30] via the QIIME (v1.8.0) software was used to cluster the high-quality sequences into operational taxonomic units (OTUs) [31], at 97% similarity. To ensure the accuracy of the subsequent analysis, the OTUs whose abundance was lower than the 0.001% of the total OTUs abundance were removed [32]. An OTU table was then constructed based on the number of sequences contained in each OTU for each sample and the sequence with the highest abundance in each OTU was selected as the representative sequence. By comparing representative OTUs sequences with reference sequences in the Human Oral Microbiome Database (HOMD) [33], the taxonomic information for each OTU was obtained, allowing us to determine the taxonomic composition of each sample at the phylum, class, order, family, genus, and species levels.

Based on the OTU table, the QIIME software was used to calculate the alpha diversity of each sample using Simpson, Chao1, ACE, and Shannon indices and rarefaction curves. Species accumulation curves and rank abundance curves were drawn with the R software. Chao1 and ACE indices were used to evaluate community richness, whereas Shannon and Simpson indices were used to evaluate both community richness and evenness.

In the taxonomic analysis, we used GraPhlAn [34] to construct and plot a taxonomic tree for determining the predominant microbiome in all samples. Nonmetric multidimensional scaling (NMDS) analysis was used to evaluate the community structure. The linear discriminant analysis effective size (LEfSe) method [35] was used to identify microbial biomarkers in each subgroup. Co-occurrence analysis was performed to demonstrate the interactions of the microbiota.

### Statistical analysis

Independent sample t test was used to compare the ages of the two groups of children, and Chi-square test to evaluate for gender. Different statistical methods were conducted based on whether the alpha diversity indices could satisfy both normality and homogeneity of variance among subgroups. One-way ANOVA or Kruskal–Wallis H test were used for the comparison of alpha diversity indices between every three subgroups, and Bonferroni test or paired comparison was used for further pair comparison. The above mentioned tests were performed with the SPSS (V20.0) software. Adonis analysis was applied for statistical testing of sample groupings in a distance matrix. The LEfSe method was implemented to compare the microbial abundances among subgroups. Spearman’s correlation coefficients among microbes were calculated using the co-occurrence analysis, performed in the 'Wu Kong' platform (https://www.omicsolution.org/wkomics/main/). *p* < 0.05 was considered statistically significant.

## Results

### Subjects, groups, and sequencing data

Thirty children underwent oral examination half of them present caries (C group), while the other half were healthy (H group, Table 1). Three samples were collected from every child (supragingival plaque of FPMs and deciduous molars, and saliva), totaling 90 samples. Based on the sample type collected, each group was divided in three subgroups, including CS, CP, CD, HS, HP, and HD. Thus, each subgroup contained 15 samples.

**Table 1:** Demographic and clinical characteristics of the 30 Chinese children

Group	Subgroup	Age^a^ (months)	Sex (male/female)	dmft^a^	dmfs^a^
Caries (n = 15)	CD, CP, CS	97.31 ± 3.22	10/5	4.07 ± 0.96	4.87 ± 1.60
Healthy (n = 15)	HD, HP, HS	97.91 ± 3.86	9/6	0	0

dmft: the number decayed, missing, or filled teeth, dmfs: the number of decayed, missing, or filled tooth surfaces

^a^Data are expressed as mean ± standard deviation

After filtering out the low-quality sequences, 418,094 high-quality sequences were obtained, with 4645 sequences per sample. The average length of the sequences was 1510 bp, 98.39% of which were distributed between 1401 and 1600 bp (Additional file 2: Fig. S1). A total of 2495 OTUs were obtained.

### Sequencing depth and sample size

Rarefaction curves showed a gentle shape, indicating that the current sequencing depth in our study was sufficient and that adequately reflected the microbial richness and evenness (Additional file 3: Fig. S2A, S2B). In the species accumulation curve, with the increase of sample size, the confidence intervals of OTUs number first increased and then gradually decreased. When the sample size was 90, the species accumulation curve was saturated (Additional file 3: Fig. S2C), indicating that the quantity of OTUs would not increase with the addition of new samples; this suggested that the sample size was sufficient for this study. The steep rank abundance curve suggested that species evenness was low across all samples (Additional file 3: Fig. S2D).

### Predominant taxon

**Fig. 1:**
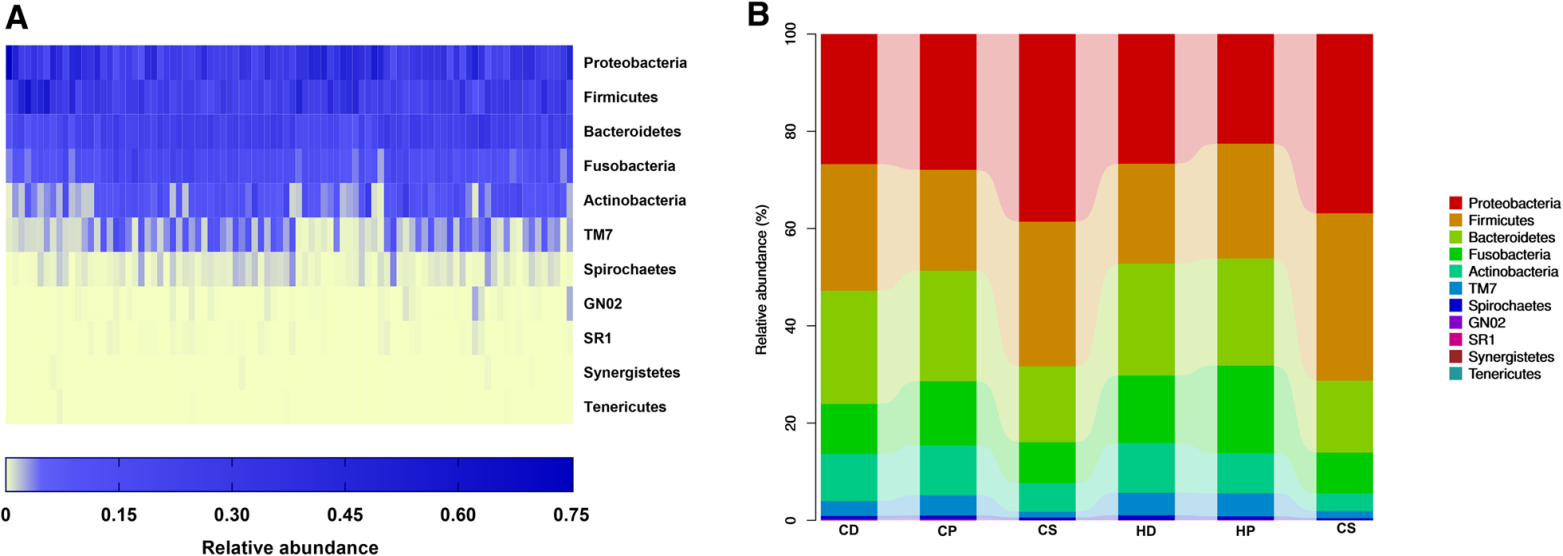
Predominant microbes at the phylum level. **A** Heat map analysis of the 90 samples. Blue indicates high relative abundance and yellow low relative abundance. **B** Bar graph analysis of the 6 subgroups. The larger the corresponding bar area, the higher the relative abundance

**Fig. 2:**
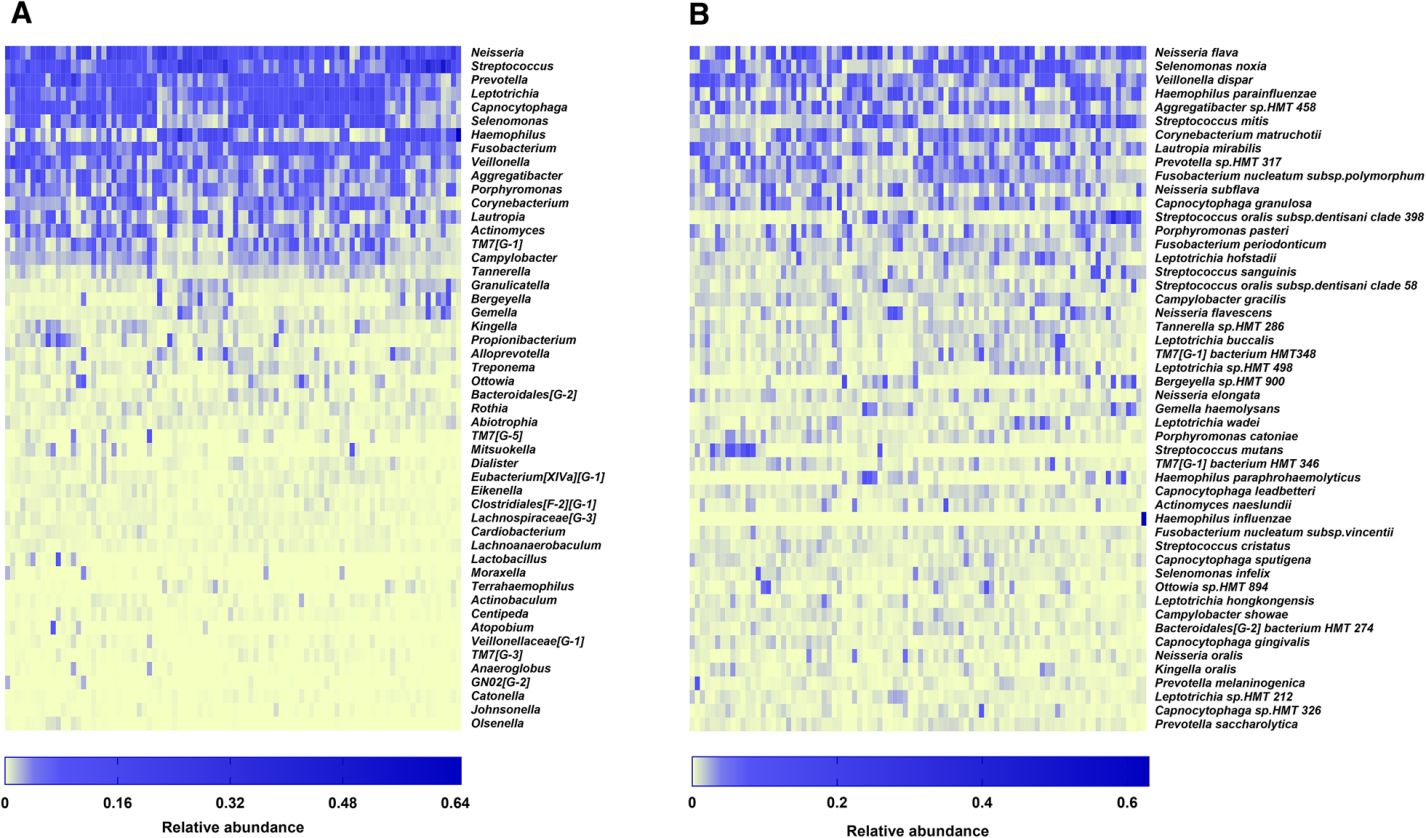
Heat maps of microbes relative abundance of the 90 samples, including the top 50 taxa **A** at the genus level and **B** at the species level

**Fig. 3:**
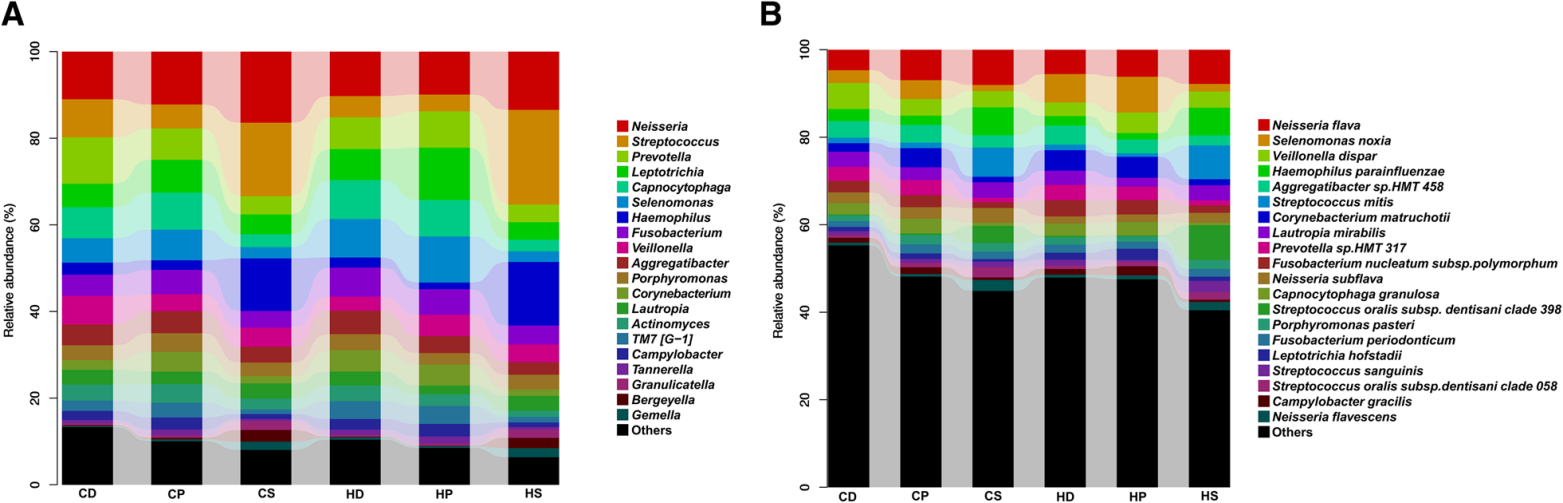
Relative abundance of the top 20 taxa in each subgroup **A** at the genus level and **B** at the species level

**Fig. 4:**
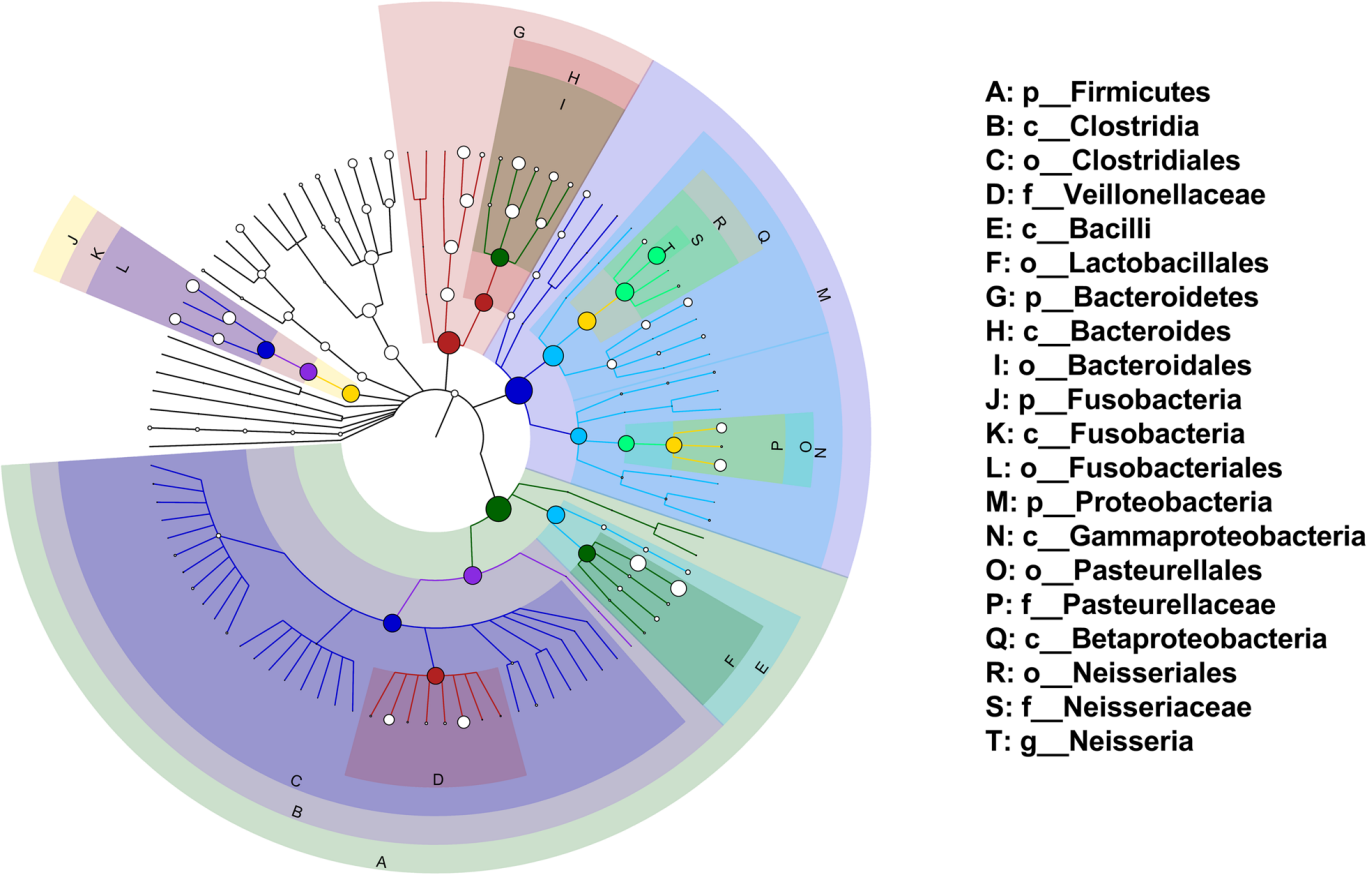
Taxonomic tree of the hierarchical relationships of all the taxa, arranged from phylum to species. Each node represents a microbiota, and the size of the node corresponds to the average relative abundance of the taxa. Twenty dominant taxa are identified in the diagram, the letters are place above the same color of their corresponding node. p, phylum; c, class; o, order; f, family; g, genus

A total of 11 phyla, 19 classes, 28 orders, 52 families, 96 genera, and 370 species of bacteria were identified, and the number of taxa in the 6 subgroups at each taxonomic level is shown in Table 2. We mainly focused on three taxonomic levels, namely, phylum, genus, and species. At the phylum level, Proteobacteria (29.9%) had the highest relative abundance, followed by Firmicutes (25.8%), Bacteroidetes (20.3%), Fusobacteria (12.0%), Actinobacteria (8.0%), and TM7 (3.2%; Fig. 1), accounting for 99.15% and 99.21% of the oral microbiota in children with and without caries, respectively. At the genus level, *Neisseria* (12.2%), *Streptococcus* (10.3%), *Prevotella* (7.0%), *Leptotrichia* (6.8%), *Capnocytophaga* (6.5%), and *Selenomonas* (6.2%) had the highest relative abundances (Figs. 2A, 3A). Of the 370 species of bacteria, the following 8 species showed high relative abundances: *Neisseria flava* (6.5%), *Selenomonas noxia* (4.2%), *Veillonella dispar* (4.2%), *Haemophilus parainfluenzae* (3.6%), *Aggregatibacter* sp. HMT 458 (3.4%), *Streptococcus mitis* (3.2%), *Corynebacterium matruchotii* (3.1%), and *Lautropia mirabilis* (3.1%; Figs. 2B, 3B). The microbes with the highest relative abundances were generally dominant. The Proteobacteria, Firmicutes, Bacteroidetes and Fusobacteria phyla, and the genus *Neisseria* were identified as the dominant microbiota by the GraPhlAn tool (Fig. 4).

**Table 2:** Number of taxa in the six subgroups at each taxonomic level

Subgroup	Phylum	Class	Order	Family	Genus	Species
CD	11	19	26	43	77	302
CP	9	17	24	39	65	255
CS	9	17	24	41	75	281
HD	11	19	25	41	71	276
HP	10	17	23	39	69	256
HS	11	19	26	48	83	280

### Spatial variation of microbiota

A total of 1,894 OTUs were obtained in the C group, and CD, CS, and CP subgroups shared 574 OTUs (Additional file 4: Fig. S3A), which accounted for 30.31% of the OTUs in the group. In the H group, 1,770 OTUs were obtained, and HD, HS, and HP subgroups shared 554 OTUs, which accounted for 31.30% of the OTUs in the group (Additional file 4: Fig. S3B).

**Fig. 5:**
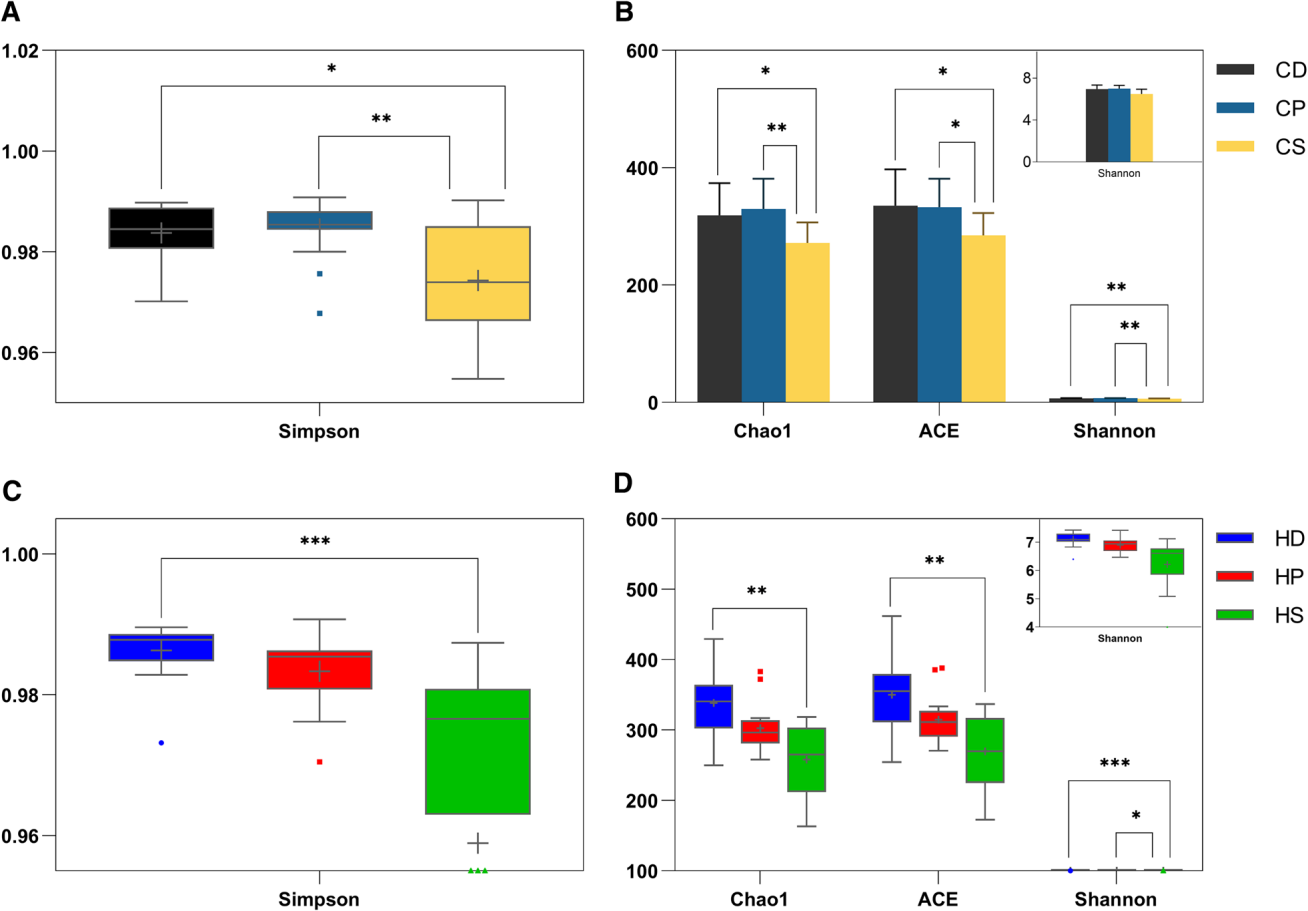
Comparison of alpha diversity indices among subgroups. **A**, **B** CD, CP, and CS subgroups. **C**, **D** HD, HP, and HS subgroups

The alpha diversity indices were calculated based on OTU data. The samples from different microniches presented different microbial alpha diversity indices. Moreover, in children with caries, the Simpson, Chao1, ACE and Shannon indices of the CS subgroup were lower than those of the CD and CP subgroups (*p* < 0.05, Fig. 5A, B). Among children without caries, the Simpson, Chao1, and ACE indices of HS subgroup were lower than those of the HD subgroup (*p* < 0.01, Fig. 5C, D), and the Shannon index of HS subgroup was lower than that of the HD and HP subgroups (*p* < 0.05, Fig. 5D). However, there was no significant difference between deciduous and permanent teeth in children with or without caries (*p* > 0.05, Fig. 5). These results indicated that the microbial richness and evenness of non-stimulating saliva were lower than that of supragingival plaque, especially in deciduous teeth.

**Fig. 6:**
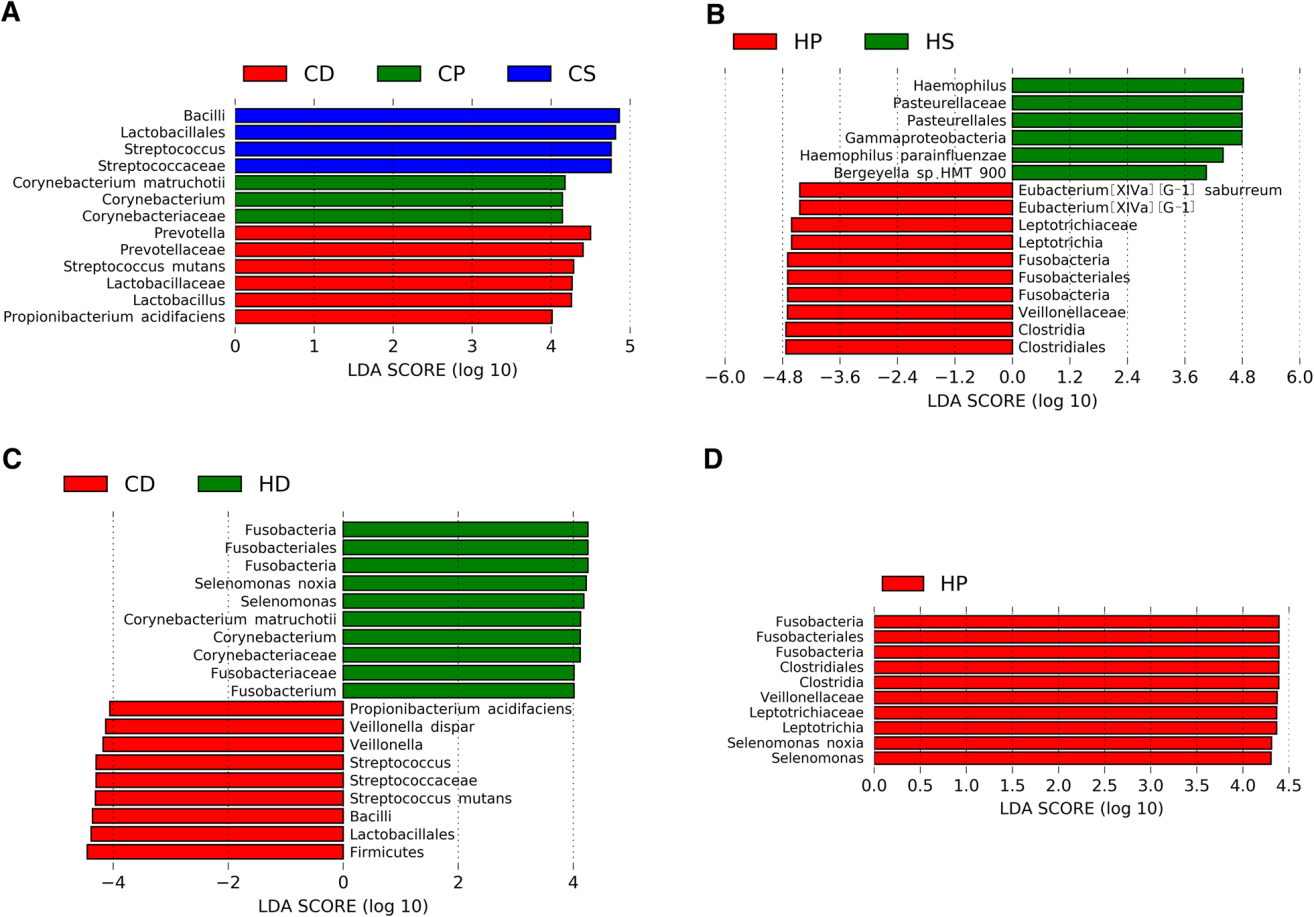
LEfSe analysis among subgroups. LEfSe analyses identified taxonomic biomarkers among the subgroups **A** CD, CP and CS; **B** HD, HP and HS; **C** CD and HD; and **D** CP and HP

The LEfSe analysis method was used to analyze the taxonomic biomarkers among subgroups, and the threshold of the logarithmic LDA score for discriminative features was set at 4.0. In similar oral environments (caries or caries-free), the abundance of the same microorganism in different spatial locations was different (*p* < 0.05). In children with caries, *Prevotella*, *Lactobacillus*, *Streptococcus mutans*, and *Propionibacterium acidifaciens* were the taxonomic biomarkers of the CD subgroup; *Corynebacterium* and *C. matruchotii* of the CP subgroup; and *Streptococcus* of the CS subgroup (Fig. 6A). In children without caries, *Eubacterium* [XIVa][G-1], *Leptotrichia*, and *Eubacterium* [XIVa][G-1] *saburreum* were the taxonomic biomarkers of the HP subgroup; *Haemophilus* and *Haemophilus parainfluenzae* of the HS subgroup (Fig. 6B); and no differences in microbial abundance were found in the HD subgroup.

**Fig. 7:**
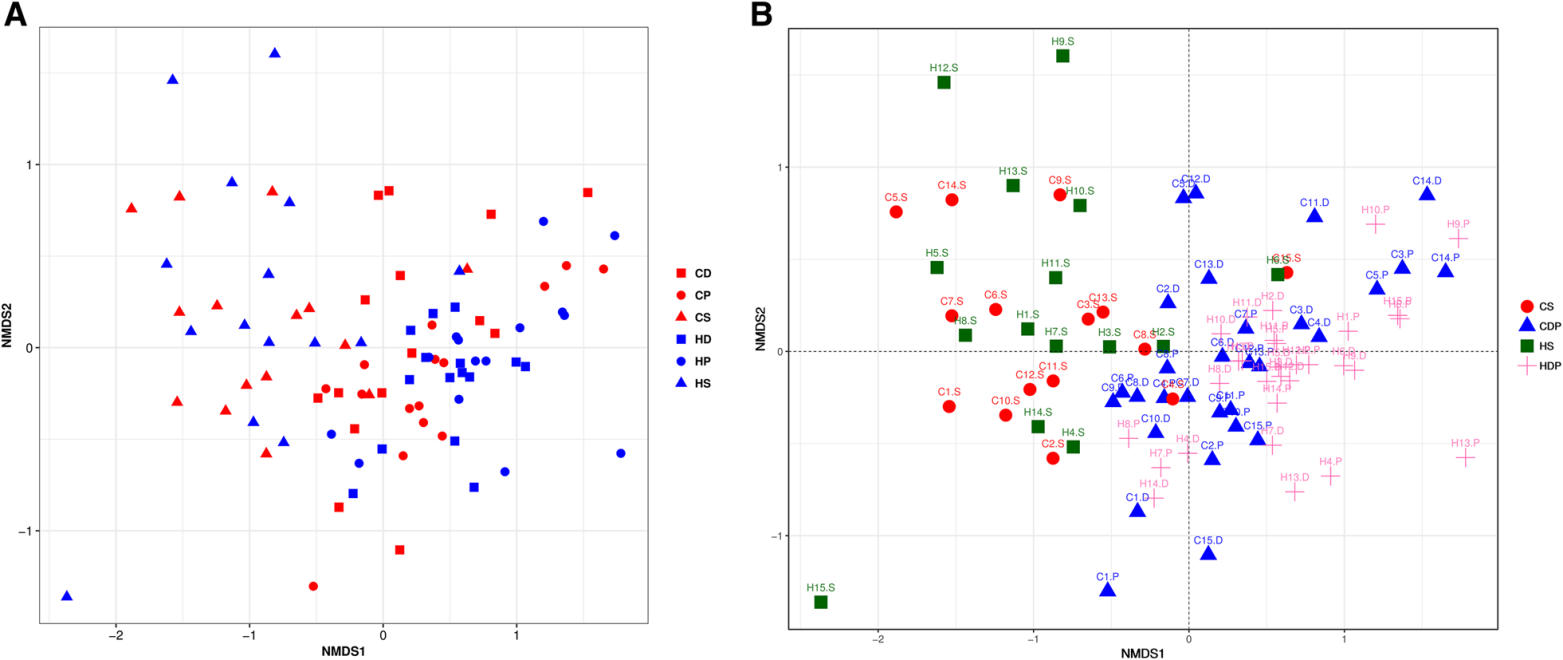
NMDS analysis based on Weighted UniFrac distances. Each sample is represented by a square/triangle/circle. **A** CD, CP, CS, HD, HP, and HS subgroups. **B** CS, CDP, HS and HDP subgroups

To evaluate the similarities and differences of the microbial community structures among subgroups, the NMDS analysis was performed, based on the Weighted UniFrac distances. As shown in Fig. 7A, saliva samples (CS and HS) were primarily scattered in the left side, whereas most plaque samples (CD, HD, CP, and HP) were distributed on the right. Since the samples of deciduous teeth and FPMs were almost distributed in the same area (Fig. 7B), the samples of deciduous teeth and PFMs with the same disease status (caries or caries-free) were mixed to represent the supragingival plaque in the Adonis analysis: the subgroup CD plus CP was CDP, and HD plus HP was HDP. The Adonis analysis demonstrated a significant difference among CS, HS, CDP, and HDP subgroups (*p* < 0.001).

### Comparison of microbial communities between children with and without caries

For children with different caries statuses, there was no significant difference in microbial alpha diversity in the same microhabitat (CD vs HD, CP vs HP, and CS vs HS). Additionally, in the plot constructed through NMDS analysis no notable separation between the subgroups (CD vs HD, CP vs HP, and CS vs HS, Fig. 7A) was observed. In the LEfSe analysis, the phylum Fusobacteria was more abundant in the HD subgroup than in the CD subgroup, whereas Firmicutes was more abundant in the CD subgroup (Fig. 6C). Furthermore, at the genus level, *Streptococcus* and *Veillonella* were considered biomarkers of the CD subgroup, and *Corynebacterium*, *Selenomonas* and *Fusobacterium* of the HD subgroup (Fig. 6C). At the species level, *S. mutans*, *V. dispar*, and *P. acidifaciens* were taxonomic biomarkers of the CD subgroup (Fig. 6C); and, in contrast, *S. noxia* and *C. matruchotii* were the taxonomic biomarkers of the HD subgroup (Fig. 6C). Compared with the CP subgroup, HP had a higher abundance of Fusobacteria, *Leptotrichia*, *Selenomonas*, and *S. noxia*. (Fig. 6D). However, we found no taxonomical biomarkers to distinguish between the CS and HS subgroups.

We selected the species with the top 100 relative abundances and calculated the Spearman's rank correlation coefficients for co-occurrence analysis, which was used to evaluate interactions among the microbes in each subgroup. There were more abundant microbes with strong associations in the caries subgroups than in the healthy subgroups (CD vs HD, CP vs HP, and CS vs HS; Additional file 5: Fig. S4). *S. mutans* and *P. acidifaciens*, both caries-associated taxa, were positively correlated in the CD and CS subgroups (Additional file 5: Fig. S4A, S4E).

## Discussion

Caries is the most common disease of the oral cavity and is mainly caused by the oral microbiota. The 16S rRNA gene sequencing is a momentous tool for identifying microorganisms. The SGS technology such as PacBio sequencing enables the obtention of highly accurate taxonomic resolution of microbial communities owing to the sequencing of full-length 16S rRNA genes [36], while the NGS platforms, which analyze part of the high-variation region (V region) of the microbial 16S rRNA gene, usually only reaches the genus level [13, 15]. Therefore, in this study we used the PacBio Sequel platform as it is capable of identifying the microbial communities with more precision than NGS, improving the analysis of microbial etiology of caries. The v1–v9 region of the 16S rRNA of the oral microorganisms was sequenced, analyzed and classified into species, obtaining a total of 370 species.

The results showed that in the same microhabitat, the microbial richness and evenness of healthy children and children with caries were similar, which was consistent with previous findings [22, 37, 38]. On the contrary, Belstrom et al. found that the alpha diversity of oral microbiota of healthy individuals was higher than that of patients with caries. Their study differed from ours in two main aspects [39], firstly, in their study the average dmfs index of the caries group was 57.1, which is more than 10 times higher than our dmfs. Accordingly, it has been shown that with the increase of tooth decay, the microbial alpha diversity decreased [15]. Secondly, the sample type collected by Belstrom et al. [39] was stimulating saliva, which is different from that used in our study. The severity of caries and sample type may influence the result of microbial alpha diversity, which needs to be verified by more in-depth studies. However, our study showed that microbial richness and evenness of saliva was lower than that of supragingival plaque in children with or without caries, which was consistent with previous studies [40, 41], suggesting that the tooth surface provided a more ideal place for microbial growth and reproduction. Taken together, the differences of microbial richness and evenness in different microniches were significant, but not in different caries statuses, suggesting that the differences in microbial diversity in spatial sites should be considered when studying microbial diversity.

Both supragingival plaque and saliva are commonly sampled for the analysis of caries. Saliva samples are easy to collect, which is suitable for children who may not cooperate with dental plaque collection. However, there are considerable differences between the supragingival plaque and saliva in terms of the microbiome [13, 41, 42], and it remains unclear whether saliva can be an alternative approach for microbial studies of caries. The results of this study showed that the microbial alpha and beta diversities between saliva and deciduous molars were different, whether in children with caries or in healthy children. In addition, the LEfSe method showed several taxonomic biomarkers in supragingival plaque samples of deciduous molars, but not in saliva samples, of children with caries and healthy children. Therefore, our findings supported that non-stimulating saliva was unsuitable for studying caries-related microorganisms. In agreement with our result, Hurley et al. found that the salivary microbiota was not sufficiently specific to identify caries risk in children [13]. In contrast, Eriksson et al. showed that the salivary microbiota was associated with cross-sectional caries prevalence [43]. These information suggest that the type of saliva collected affects the experimental results, since both our study and that of Hurley et al. analyzed non-stimulating saliva [13], whereas Eriksson et al. used stimulating saliva [43].

In deciduous teeth plaque, *Fusobacterium*, a type of asaccharolytic bacteria [44], was the more abundant genera in healthy children when compared with the caries group. Similarly, a previous study demonstrated that this microorganism show higher bacterial activity on healthy surfaces than on caries lesions, suggesting that *Fusobacterium* may influence tooth health [45]. Furthermore, we identified *Streptococcus* and *Veillonella* as two microbial biomarkers in deciduous molar plaque of children with caries, compared with the corresponding healthy subgroup. Consistent with this, Dzidic et al. found that *Streptococcus* and *Veillonella* inhabited the oral cavity during the first 3–6 months of life, designating these bacteria as early colonizers [46]. Indeed, these bacteria are generally found in children with ECC [13, 22, 47], which was also found in the decayed deciduous teeth in our study.

At the species level, *P. acidifaciens* not only was more abundant in healthy children than children with caries in terms of deciduous teeth plaque, but was also identified as a caries-specific bacterium, absent in healthy children, and was detected in 13 of the children with caries. *P. acidifaciens* showed a higher abundance in individuals with caries in previous studies [48–50]. In 2009, Downes and Wade isolated *P. acidifaciens* from the human oral cavity for the first time [51]. Additionally, Obata et al. found that *P. acidifaciens* could strongly combine with collagen (dentine contains approximately 20% organic matter, including collagen) and could produce acid in a low-pH environment, features that could contribute to the occurrence of dentine caries [52].

*Selenomonas noxia* was the second most abundant species in all samples. The LEfSe analysis also showed that *S. noxia* had a higher abundance in the deciduous teeth plaque of healthy children than children with caries, consistent with a study by Preza et al. [53]. *S. noxia*, a known periodontal pathogen, was isolated, cultured, and identified by Moore and his team in human periodontal pockets in 1987 [54]. Moreover, this microorganism is responsible for the loss of attachment in periodontitis [55] and is present at higher levels in patients with periodontitis than in healthy individuals [56, 57]. Accordingly, the increased or decreased abundance of *S. noxia* may lead to different oral diseases. Additionally, *S. noxia* was identified as a microbial biomarker in the FPM plaque of healthy children in our study. The prevention of FPM damage is critical, as they are essential for establishing occlusion and are the most important teeth for mastication. However, since the FPMs are the earliest permanent teeth to erupt, they are the most prone to caries. Moreover, the prevalence of caries in the FPMs is closely related to the presence of deciduous caries. In 1959, Bruszt et al. recorded the caries statuses of 97 children at 5 and 11 years of age and found that 94.6% of children with deciduous caries had permanent caries 6 years later [58]. In recent years, both cross-sectional and longitudinal studies have shown that deciduous tooth caries is a risk factor for caries in permanent molars (particularly FPMs) [24–26]. Nevertheless, no microbiological studies have evaluated the correlations between deciduous and permanent molar caries. Our findings revealed that reduction of *S. noxia* occurs in healthy FPMs of children with deciduous caries, suggesting that it could be associated with increased caries risk in FPMs in children with deciduous caries.

Of the hundreds of oral bacteria, *S. mutans* is most frequently associated with dental caries and is the primary pathogenic bacteria of caries due to its ability to colonize the oral cavity, form a firm biofilm on the surface of the teeth, survive and reproduce in an acidic environment, and continue to produce acid [59, 60]. In our study, *S. mutans* was a microbial biomarker in deciduous molar plaque of children with caries, compared with healthy children, which indicates that this bacteria was closely associated with caries. However, neither all children with caries tested positive for *S. mutans*, nor were all healthy children negative for the microorganism. We found that it was present in 14 children with caries and that *V. dispar*, another microbial biomarker in deciduous teeth plaque of children with caries, was present in *S. mutans*-negative individuals, suggesting that caries was not caused by a single bacterium but by a combination of multiple bacteria.

Co-occurrence analysis showed potential interactions among the oral microbiota. Compared with healthy subgroups, there were more abundant correlation pairs of microorganisms in the subgroups of the caries group, indicating that the microbial community have more complex relationships in children with caries. *Streptococcus gordonii* and *TM7[G-1] bacterium HMT 347* were positively correlated in the CD subgroup but showed no correlation in the HD subgroup. *Neisseria subflava* and *TM7[G-1] bacterium HMT 347* were negatively correlated in the HD subgroup but showed no correlation in the CD subgroup. These findings suggested that the same organisms interacted differently in different environments (caries or caries-free). Notably, in a previous study, biofilm formation by *P. acidifaciens* was found to be inhibited by *S. mutans* [52]. Contrary to this result, we found a positive correlation between *S. mutans* and *P. acidifaciens* in the co-occurrence analysis.

The time necessary for caries development is greatly variable; thus, as this study was a cross-sectional study it lacked longitudinal measurements of microbial changes. In a future longitudinal study, oral examination and sampling should be performed at the beginning and the end of each year of the study. The caries status of the FPMs should be recorded from the healthy state until caries formation, and the collected supragingival plaque of the FPMs should be sequenced to identify the FPMs caries-related microbiome. Then, animal experiments should be performed to determine if the previously identified caries-related microbes can actually cause caries. If the results are promising, it would be possible to screen children at high risk of caries development by detecting the microbiome of their FPMs, and also to focus on prevention, for example, through pit and fissure sealing, regular oral examination and fluorine application.

## Conclusion

In this study, the composition and structure of the oral microbiome were analyzed from the perspectives of different microniches and caries statuses. In the same caries status, the distribution of microbial is spatially different, which should be taken into account during sampling when studying oral microbiota. In the same sample type, *S. mutans*, *V. dispar*, and *P. acidifaciens* were highly correlated with caries of deciduous teeth, whereas no taxonomic biomarkers were found in saliva samples, suggesting that dental plaque was more representative than saliva for identifying the caries-related microorganisms. Further studies are necessary to determine the reason for the correlation between *S. mutans* and *P. acidifaciens*. Additionally, *S. noxia* was more abundant in the supragingival plaque of FPMs in healthy children, compared with that of the children with caries. To the best our knowledge, this study is the first to provide a reference on the microbiological differences in healthy FPMs in different caries statuses, thereby establishing a microbial basis for prevention of caries in FPM.

## Supplementary Information

**Additional file 1: Table S1**. Barcode sequence of 90 samples.

**Additional file 2: Fig. S1**. Length distributions of high-quality sequences. 74.36% of the high-quality sequences were distributed between 1,501 and 1,600 bp; and 24.03% were distributed between 1,401 and 1,500 bp.

**Additional file 3: Fig. S2**. Curve charts for 90 samples. (A) Rarefaction curves based on Shannon index, each curve represents one sample. (B) Rarefaction curves based on OTUs, each curve represents one sample. (C) Species accumulation curves. The blue-shaded areas represent confidence intervals of OTUs number which was determined. (D) Rank abundance distribution curves. Each broken line represents the OTU abundance distribution of one sample.

**Additional file 4: Fig. S3**. Venn Diagrams representing the number of OTUs among every three subgroups. The numbers in each circle show the number of OTUs found in each subgroup, and the overlap represents the shared OTUs. (A) CD, CS, and CP subgroups. (B) HD, HS, and HP subgroups.

**Additional file 5: Fig. S4**. Interactions among the microbes in each subgroup (|ρ| > 0.8 and *p* < 0.05). Each circle or square represents one microbe, the red line represents a positive correlation between two microbe species, and the blue line a negative correlation between two microbe species. (A) CD subgroup. (B) HD subgroup. (C) CP subgroup. (D) HP subgroup. (E) CS subgroup. (F) HS subgroup.

## Data Availability

Raw sequencing data have been deposited in the National Center for Biotechnology Information (NCBI) Sequence Read Archive (SRA) database (accession number PRJNA725075). Patient data are available from the corresponding author on reasonable request.

## Abbreviations

NGS: Next-generation sequencing
PCR: Polymerase chain reaction
FPM: First permanent molar
CCS: Circular consensus sequencing
ZMW: Zero-mode waveguide
OTU: Operational taxonomic unit
HOMD: Human Oral Microbiome Database
NMDS: Nonmetric multidimensional scaling
LDA: Linear discriminant analysis
LEfSe: Linear discriminant analysis effective size
PacBio: Pacific Biosciences
ECC: Early childhood caries
rRNA: Ribosomal RNA
TGS: Third-generation sequencing
