# Supplementary material for: The impact of caries status on supragingival plaque and salivary microbiome in children with mixed dentition: a cross-sectional survey
Source: BMC Oral Health. 2021 Jun 25;21:319. doi: 10.1186/s12903-021-01683-0 (PMC8229229; doi:10.1186/s12903-021-01683-0)
Supplement: Supplementary file 1 — Additional file 1: Table S1. Barcode sequence of 90 samples. [file 12903_2021_1683_MOESM1_ESM.docx]

**Table S1. Barcode sequence of 90 samples**

| Sample ID | barcode |
| --- | --- |
| 01C.D | GCGCTCTGTGTGCAGC |
| 02C.D | TCATGAGTCGACACTA |
| 03C.D | TATCTATCGTATACGC |
| 04C.D | ATCACACTGCATCTGA |
| 05C.D | ACGTACGCTCGTCATA |
| 06C.D | TGTGAGTCAGTACGCG |
| 07C.D | AGAGACACGATACTCA |
| 08C.D | CTGCTAGAGTCTACAG |
| 09C.D | AGCACTCGCGTCAGTG |
| 10C.D | TCATGCACGTCTCGCT |
| 11C.D | AGAGCATCTCTGTACT |
| 12C.D | CGCATCGACTACGCTA |
| 13C.D | CGTAGCGTGCTATCAC |
| 14C.D | ATGCTGATGACTGCGA |
| 15C.D | TGCGTGAGCTGTACAT |
| 01C.P | CGATCATCTATAGACA |
| 02C.P | CGACGTATCTGACAGT |
| 03C.P | CACGTCACTAGAGCGA |
| 04C.P | TGTCGCAGCTACTAGT |
| 05C.P | CATACGCTGTGTAGCA |
| 06C.P | AGTCGCATGACTGTGT |
| 07C.P | CAGTACTGCACGATCG |
| 08C.P | GTGCTGAGCATCAGAC |
| 09C.P | CACTGATCGATATGCA |
| 010C.P | TACAGTGTCTGCTGCG |
| 011C.P | TACAGATAGTGTAGCG |
| 012C.P | TCGTAGAGCTCGAGAC |
| 013C.P | GAGCTGCGCACTCGAT |
| 014C.P | GCGATGTCGCTATGTG |
| 015C.P | CGAGAGTCAGCGCATA |
| 01C.S | TCACGATGAGCACGTA |
| 02C.S | GACTGAGATCATGATC |
| 03C.S | ACGACATGATACTGCT |
| 04C.S | ATACAGCACAGATGTG |
| 05C.S | ACAGTCGATATCTCTC |
| 06C.S | GCTCGATCACATGACG |
| 07C.S | GTCGTACACGTGCGAC |
| 08C.S | ACTCATATCTAGAGTG |
| 09C.S | ACTGATCTGTCGCGCT |
| 10C.S | CACTAGCTCTGACTAC |
| 11C.S | GCTGTCATGTACTAGC |
| 12C.S | TATACATACACGCACT |
| 13C.S | TGTGACGACGCGTCTC |
| 14C.S | GACGTGAGCATGCACT |
| 15C.S | CTCGATACGTGTAGCT |
| 01H.D | GTGTCTAGACAGCTGT |
| 02H.D | GATGCATGCGTACGCA |
| 03H.D | TATCAGAGCAGCGATG |
| 04H.D | AGAGTACTACATATGA |
| 05H.D | CGTGTGCATAGATCGC |
| 06H.D | ATGTATCTCGACTGCA |
| 07H.D | GACTCGACGCAGAGTC |
| 08H.D | CGATGACGTCGCTGTA |
| 09H.D | CACACGTAGTCTGCGC |
| 10H.D | GCTGTATCGCAGAGAC |
| 11H.D | CGAGCTATCTCATACT |
| 12H.D | CATGAGTACTCGTCGC |
| 13H.D | CAGCGACTGTGATACT |
| 14H.D | TGTCGCATCATATGAT |
| 15H.D | GCTGTGATCTACGTCT |
| 01H.P | TCATGCACGTCTCGCT |
| 02H.P | AGAGCATCTCTGTACT |
| 03H.P | CGCATCGACTACGCTA |
| 04H.P | CGTAGCGTGCTATCAC |
| 05H.P | ATGCTGATGACTGCGA |
| 06H.P | TGCGTGAGCTGTACAT |
| 07H.P | CGATCATCTATAGACA |
| 08H.P | CGACGTATCTGACAGT |
| 09H.P | CACGTCACTAGAGCGA |
| 10H.P | TGTCGCAGCTACTAGT |
| 11H.P | CATACGCTGTGTAGCA |
| 12H.P | AGTCGCATGACTGTGT |
| 13H.P | CAGTACTGCACGATCG |
| 14H.P | GTGCTGAGCATCAGAC |
| 15H.P | CACTGATCGATATGCA |
| 01H.S | ATACAGCACAGATGTG |
| 02H.S | ACAGTCGATATCTCTC |
| 03H.S | GCTCGATCACATGACG |
| 04H.S | GTCGTACACGTGCGAC |
| 05H.S | ACTCATATCTAGAGTG |
| 06H.S | ACTGATCTGTCGCGCT |
| 07H.S | CACTAGCTCTGACTAC |
| 08H.S | GCTGTCATGTACTAGC |
| 09H.S | TATACATACACGCACT |
| 10H.S | TGTGACGACGCGTCTC |
| 11H.S | GACGTGAGCATGCACT |
| 12H.S | CTCGATACGTGTAGCT |
| 13H.S | GTGTCTAGACAGCTGT |
| 14H.S | GATGCATGCGTACGCA |
| 15H.S | TATCAGAGCAGCGATG |
