# Supplementary material for: The impact of caries status on supragingival plaque and salivary microbiome in children with mixed dentition: a cross-sectional survey
Source: BMC Oral Health. 2021 Jun 25;21:319. doi: 10.1186/s12903-021-01683-0 (PMC8229229; doi:10.1186/s12903-021-01683-0)
Supplement: Supplementary file 2 — Additional file 2: Fig. S1. Length distributions of high-quality sequences. 74.36% of the high-quality sequences were distributed between 1,501 and 1,600 bp; and 24.03% were distributed between 1,401 and 1,500 bp. [file 12903_2021_1683_MOESM2_ESM.docx]

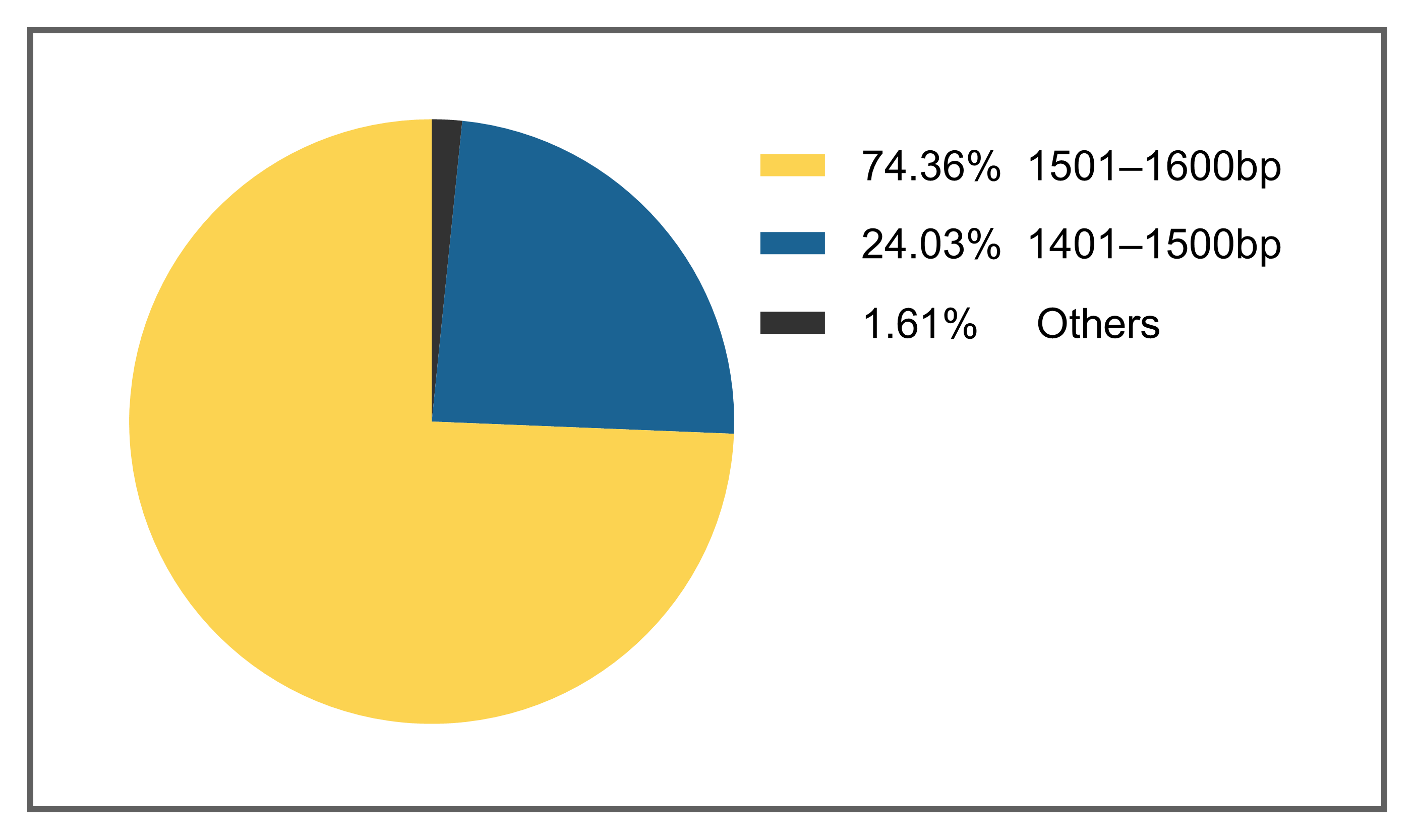


**Figure S1.** Length distributions of high-quality sequences. 74.36% of the high-quality sequences were distributed between 1,501 and 1,600 bp; and 24.03% were distributed between 1,401 and 1,500 bp.
