# Supplementary material for: The impact of caries status on supragingival plaque and salivary microbiome in children with mixed dentition: a cross-sectional survey
Source: BMC Oral Health. 2021 Jun 25;21:319. doi: 10.1186/s12903-021-01683-0 (PMC8229229; doi:10.1186/s12903-021-01683-0)
Supplement: Supplementary file 3 — Additional file 3: Fig. S2. Curve charts for 90 samples. (A) Rarefaction curves based on Shannon index, each curve represents one sample. (B) Rarefaction curves based on OTUs, each curve represents one sample. (C) Species accumulation curves. The blue-shaded areas represent confidence intervals of OTUs number which was determined. (D) Rank abundance distribution curves. Each broken line represents the OTU abundance distribution of one sample. [file 12903_2021_1683_MOESM3_ESM.docx]

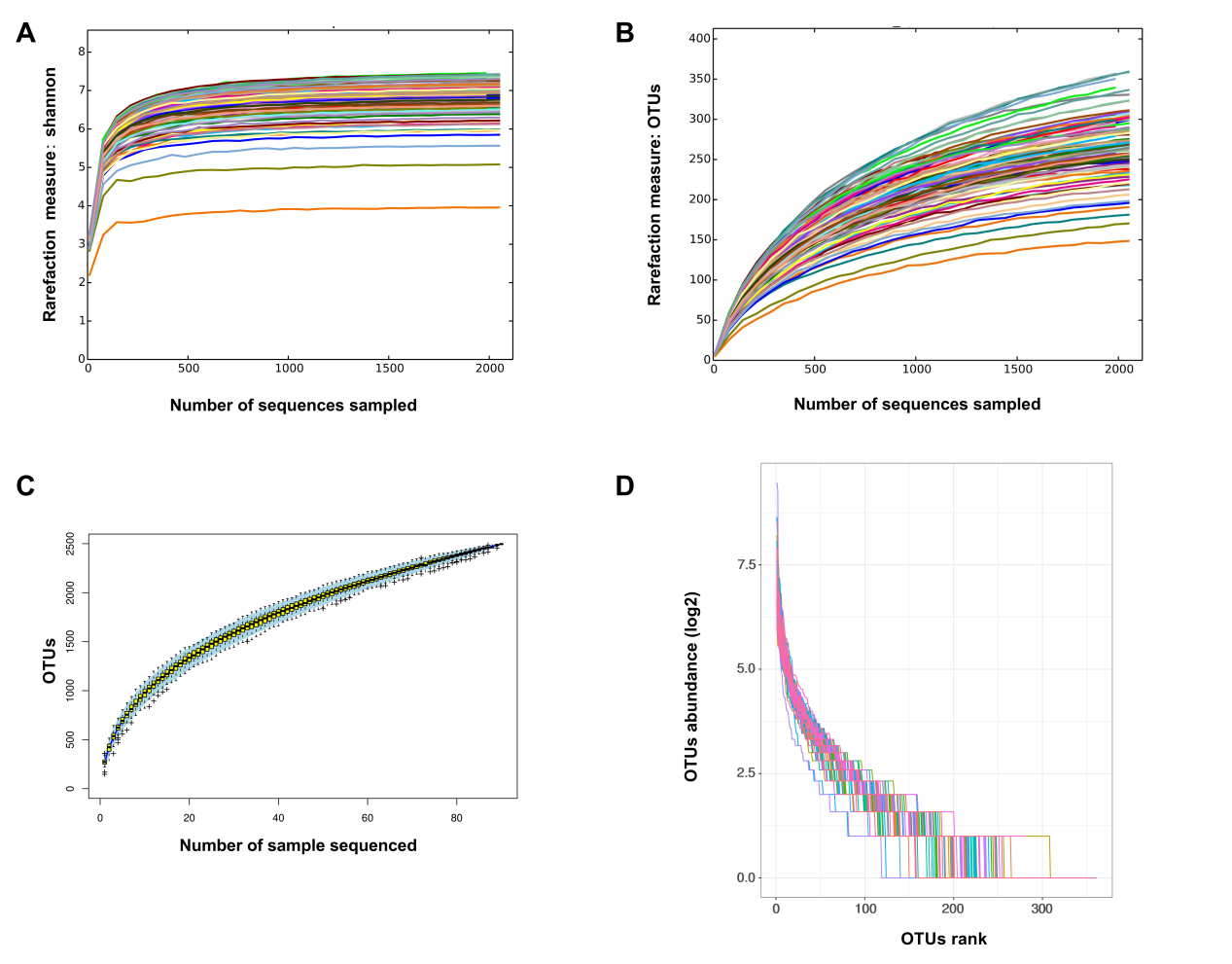


**Figure S2.** Curve charts for 90 samples. (A) Rarefaction curves based on Shannon index, each curve represents one sample. (B) Rarefaction curves based on OTUs, each curve represents one sample. (C) Species accumulation curves. The blue-shaded areas represent confidence intervals of OTUs number which was determined. (D) Rank abundance distribution curves. Each broken line represents the OTU abundance distribution of one sample.
