# Supplementary material for: The impact of caries status on supragingival plaque and salivary microbiome in children with mixed dentition: a cross-sectional survey
Source: BMC Oral Health. 2021 Jun 25;21:319. doi: 10.1186/s12903-021-01683-0 (PMC8229229; doi:10.1186/s12903-021-01683-0)
Supplement: Supplementary file 4 — Additional file 4: Fig. S3. Venn Diagrams representing the number of OTUs among every three subgroups. The numbers in each circle show the number of OTUs found in each subgroup, and the overlap represents the shared OTUs. (A) CD, CS, and CP subgroups. (B) HD, HS, and HP subgroups. [file 12903_2021_1683_MOESM4_ESM.docx]

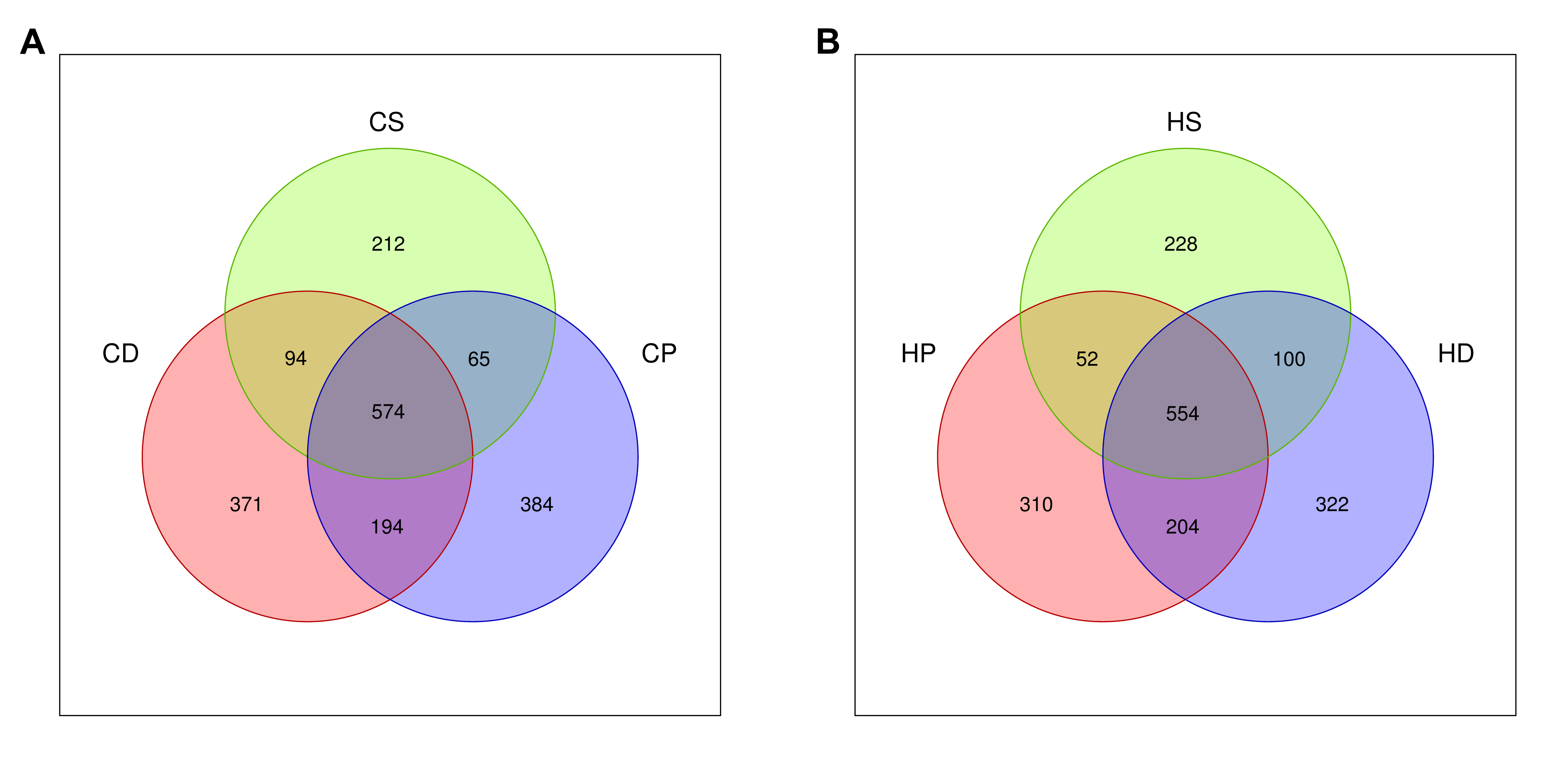


**Figure S3.** Venn Diagrams representing the number of OTUs among every three subgroups. The numbers in each circle show the number of OTUs found in each subgroup, and the overlap represents the shared OTUs. (A) CD, CS, and CP subgroups. (B) HD, HS, and HP subgroups.
