# Supplementary material for: The impact of caries status on supragingival plaque and salivary microbiome in children with mixed dentition: a cross-sectional survey
Source: BMC Oral Health. 2021 Jun 25;21:319. doi: 10.1186/s12903-021-01683-0 (PMC8229229; doi:10.1186/s12903-021-01683-0)
Supplement: Supplementary file 5 — Additional file 5: Fig. S4. Interactions among the microbes in each subgroup (|ρ| > 0.8 and p < 0.05). Each circle or square represents one microbe, the red line represents a positive correlation between two microbe species, and the blue line a negative correlation between two microbe species. (A) CD subgroup. (B) HD subgroup. (C) CP subgroup. (D) HP subgroup. (E) CS subgroup. (F) HS subgroup. [file 12903_2021_1683_MOESM5_ESM.docx]

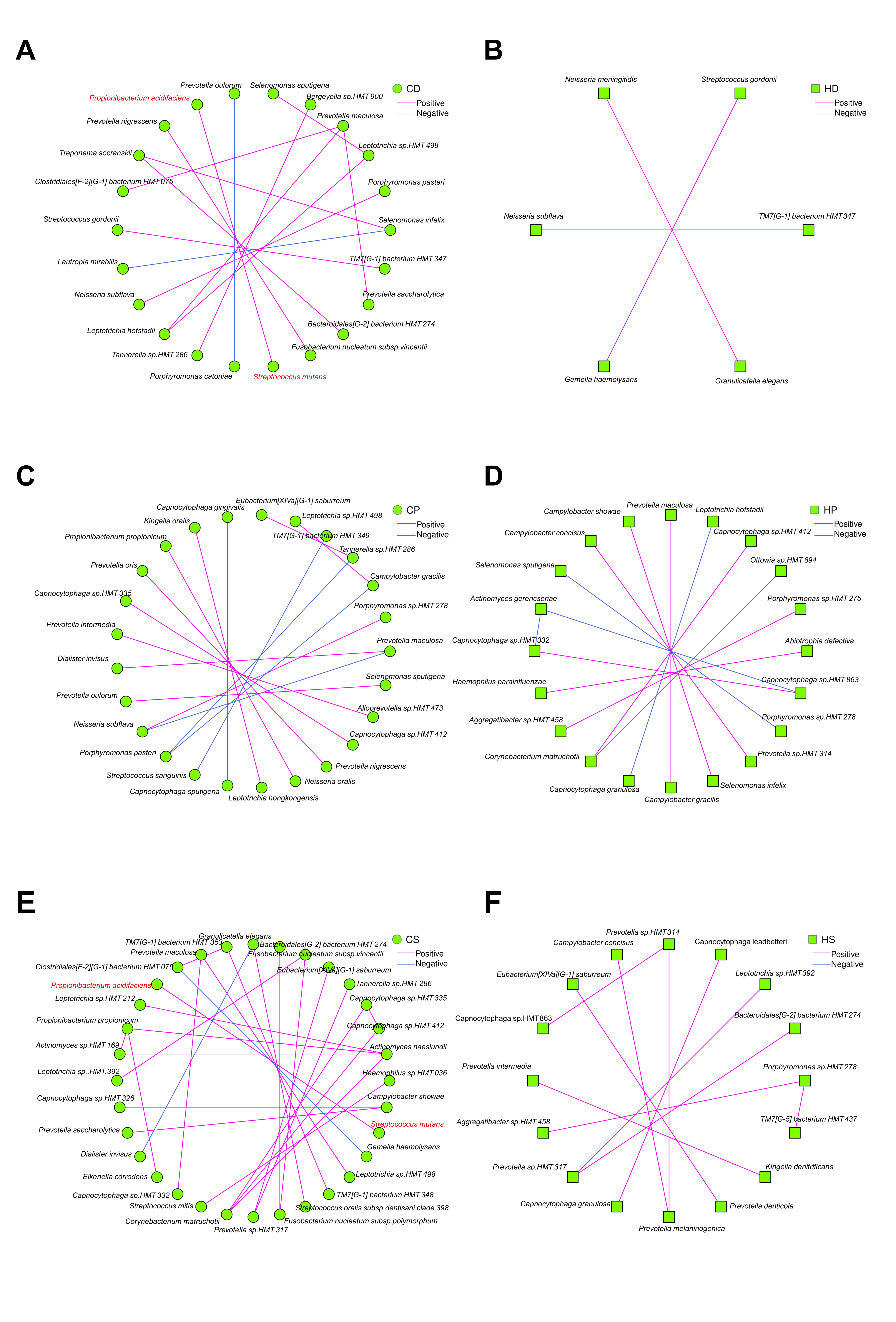


**Figure S4.** Interactions among the microbes in each subgroup (|ρ|>0.8 and *p*<0.05). Each circle or square represents one microbe, the red line represents a positive correlation between two microbe species, and the blue line a negative correlation between two microbe species. (A) CD subgroup. (B) HD subgroup. (C) CP subgroup. (D) HP subgroup. (E) CS subgroup. (F) HS subgroup.
